# Visual Perception of Procedural Textures: Identifying Perceptual Dimensions and Predicting Generation Models

**DOI:** 10.1371/journal.pone.0130335

**Published:** 2015-06-24

**Authors:** Jun Liu, Junyu Dong, Xiaoxu Cai, Lin Qi, Mike Chantler

**Affiliations:** 1 Department of Computer Science and Technology, Ocean University of China, 238 Songling Road, Qingdao, Shandong, China; 2 Science and Information College, Qingdao Agricultural University, 700 Changcheng Road, Qingdao, Shandong, China; 3 Computer Science Department, Heriot-Watt University, Edinburgh, Scotland; University of Chicago, UNITED STATES

## Abstract

Procedural models are widely used in computer graphics for generating realistic, natural-looking textures. However, these mathematical models are not perceptually meaningful, whereas the users, such as artists and designers, would prefer to make descriptions using intuitive and perceptual characteristics like "repetitive," "directional," "structured," and so on. To make up for this gap, we investigated the perceptual dimensions of textures generated by a collection of procedural models. Two psychophysical experiments were conducted: free-grouping and rating. We applied Hierarchical Cluster Analysis (HCA) and Singular Value Decomposition (SVD) to discover the perceptual features used by the observers in grouping similar textures. The results suggested that existing dimensions in literature cannot accommodate random textures. We therefore utilized isometric feature mapping (Isomap) to establish a three-dimensional perceptual texture space which better explains the features used by humans in texture similarity judgment. Finally, we proposed computational models to map perceptual features to the perceptual texture space, which can suggest a procedural model to produce textures according to user-defined perceptual scales.

## Introduction

### Texture Perception

Studies of texture perception have great significance for image understanding, data visualization and image retrieval. In neuroscience and psychophysics, research mainly focuses on neural processes involved in visual perception of textures, with a great amount of effort made on understanding the mechanism of texture detection and segregation. Pioneering works on texture perception investigated human’s discriminability of artificial texture pairs [1–4]. The human vision system is powerful in extracting features for texture discrimination, and this feature extraction mechanism works well for all textures. Thus, two challenging questions arise intuitively: what textural features are used and how are they used by humans in texture perception?

Over the last few decades, efforts have been made on extracting textural features [5–9], and some of them were thought to be representations of perceptual features, such as repetitiveness, directionality, coarseness, and so on [10–17]. The experimental results showed certain correspondence between these features and human perception. However, the fore-mentioned perceptual features were intuitively or empirically selected; in general, no robust models were available for effectively extracting textural features in consistency with human perception.

It is commonly known that colors can be represented in a variety of three-dimensional spaces, for example, the HSI and RGB color space. Inspired by studies in color perception, researchers attempted to identify a multi-dimensional texture space so that the mechanism underlying texture perception can be revealed. The perceptual texture space (PTS) is in analogue to a color space; once the dimensions have been identified, they can be used as a standard representation for texture and for perceptual similarity judgment.

The pioneering study of developing such perceptual texture space was conducted by Rao and Lohse [18]. Twelve perceptual features aiming to capture different aspects of texture were elaborately selected for psychophysical experiments. A three-dimensional texture space was established based on the data obtained through grouping and rating experiments. The dimensions of the space were identified to be related to the most important three perceptual features, i.e. repetitiveness, contrast/directionality and coarseness/complexity. Heaps and Handle [19] did similar experiments using natural textures. In contrast, they found that perceived texture similarity was context-dependent and the perceptual meaning of the PTS dimensions was difficult to name. It might be argued that similarity judgments were affected by material characteristics.

There are several studies that explore the interactions among different material properties. Fleming et al [20] studied material perception by investigating the relationship between material classes and perceptual qualities in both visual and semantic domains. Results suggested that ratings of various material properties could be used to identify material classes. Ho et al [21] used a conjoint-measurement design to assess how the surface roughness and glossiness affected perception of each other. Oliva et al [22] tried to identify the perceptual dimension of visual complexity of scenes composed of objects, textures and colors. They found that there existed a multi-dimensional space with a set of perceptual dimensions, although the dimensions were modulated by task constraints. These works suggest that identifying the perceptual texture dimensions can help us to understand the human perception mechanism not only towards texture, but also all visual stimuli.

Subspace transformation techniques are commonly used to obtain the embedded dimensions in a data set [23, 24]. As a linear approach, principal component analysis (PCA) and multidimensional scaling (MDS) had been broadly used in revealing the mechanisms underlying color and texture perception [18, 19, 25, 26], as well as visual object classification [20, 27–29]. However, many data sets contained essentially nonlinear structures that were invisible to MDS; thus the true structure of high dimensional data may not be revealed. In contrast, the isometric feature mapping algorithm (Isomap) is one of several widely used non-linear methods that can discover low-dimensional representations for high-dimensional data. In Isomap, geodesic distances on a weighted graph are incorporated with the MDS. It guarantees asymptotically to recover the true dimensionality of nonlinear manifold and provide globally optimal solution. Oliva et al [22] applied Isomap to a dissimilarity matrix obtained from grouping experiments to construct a multidimensional space for visual complexity representation.

A reliable perceptual texture space can accommodate a metric that can be effectively used for perceived similarity measurement; pairwise distances between points in this perceptual space represent the degree of texture similarity [25, 30]. Thus, it is important to model the mapping from different textural features to perceptual dimensions in the PTS. In [30–32], Support Vector Machines (SVM) were used to construct a mapping from computational features to perceptual space. It was also reported that such kind of mapping in the PTS improved perceptual consistency.

### Procedural Textures

Procedural texture models have the advantage that different texture with arbitrary sizes can be effectively produced by simply varying model parameters [33]. In the past decades, various approaches have been proposed [33, 34] to create realistic representation of natural elements such as wood, marble, metal, stone and many other material appearances. For example, Perlin noise [34, 35] is often used to simulate fire, smoke, clouds, or visual effects resembling complex natural phenomena in movies.

The majority of early studies in texture perception utilized artificial textures composed of micropatterns (e.g. dots, line segments, L’s, T’s and X’s) that were placed in a regular or random way. However, they were not or could not be thought of as originating from our natural environment. For the reason that human visual cortex is likely to be nonlinear and tuned to natural stimuli, results based on these artificial textures are not guaranteed to be consistent with human visual perception [36]. Accordingly, natural textures(e.g. Brodatz textures [37]) were utilized in recent studies on texture perception. However, as far as visual perception is concerned, natural textures always contain objects or surfaces that can be easily interpreted by human subjects as context and material description. In addition, most datasets were captured under uncontrolled viewing and illumination conditions, and it has been proved that changes in illumination condition affect the appearance of textured surfaces and could cause significant variations in observers’ visual perceptions [36, 38].

In contrast, synthesized images resembling natural-like materials have been explored for material perception. For instance, Matusik et al [39] proposed a perceptual model based on measured reflectance properties (BRDFs) to generate novel BRDFs with expected visual qualities. Weinmann used a BTF material database to produce synthetic images under different viewing and illumination conditions to simulate real-world materials [40]. Compared with natural or synthetic images used in previous work, procedural textures provide a better solution which balances in natural-like appearance and parameterization. An arbitrary number of textures can be produced by procedural texture models with controlled lighting.

Identifying perceptual features of procedural texture is important, because humans naturally use perceptual characteristics to describe textures, such as “repetitive”, “directional” and “highly structured”. They do not have or need not have knowledge on computational features, which may be perceptually meaningless, although some computational features, such as statistical features proposed by Portilla and Simoncelli [7], have been successfully used for texture classification and discrimination in computer vision. With perceptual features, even inexperienced computer graphics users or artists can effortlessly describe a texture generation model.

Procedural models are different in terms of texture appearances they can generate. Unless one is familiar with procedural models and their output, it is difficult to predict which models can produce which types of texture. Our previous study has shown that a set of relevant perceptual features could be used to discriminate near-regular texture classes as human perceived; nevertheless, they are not good enough for discriminating random textures [41]. It is not practical to simply map certain perceptual characteristics to specific procedural models, e.g. using a look-up table. The solution for selecting a procedural model according to user-defined perceptual description is yet to be found.

Our work focused on identifying perceptual dimensions of procedural textures, and how to automatically find an appropriate procedural model which can generate textures with perceptual features as user defined. To achieve this, we firstly generated a dataset of procedural textures, and then conducted two psychophysical experiments: grouping and rating. The data was analyzed using HCA and SVD to obtain model clusters and corresponding features. Secondly, we derived a perceptual texture space (PTS) by applying Isomap to the dissimilarity matrix obtained from the grouping experiment. Then we identified the perceptual dimensions according to the correlation between the dimensions of PTS and the twelve perceptual features introduced by Rao et al. We compared these perceptual features with the PTS features in terms of the classification performance, i.e. they were used as features for classifying each sample into corresponding generation models. This allowed us to determine whether the twelve perceptual features were sufficient to discriminate different categories of textures. Furthermore, three models were trained to map the 12-dimensional perceptual features to three dimensions in the perceptual texture space respectively. This three-dimensional PTS provided more convincing results for classification and discrimination of visual texture. With the PTS, we are able to predict a procedural texture generation model that can produce textures with specified perceptual scales.

## Methods

### Ethics statement

The experimental procedure was approved by the IRB of the Ocean University of China. The subjects signed informed consent and had the right to quit the experiment at any time.

### Subjects

In total, seventy-eight subjects with normal or correct to normal vision (undergraduate and graduate students in the university) participated in the two psychophysical experiments. Twenty graduate students participated in the first experiment and fifty-eight students participated in the second experiment. All subjects had no knowledge of procedural models and were unaware of the purpose of the psychophysical experiments.

### Stimuli

Twenty-three representative procedural texture generation models, including mathematical models and filtering/post-processing models, were selected as in [41]. Varying model parameters produced dramatically different textures. The details of the models were summarized below.

1. Cellular Automaton (Forest fire model) (CA (Forest fire model)) [42–44]
2. Cellular Automaton (Surface tension model) (CA (Surface tension model))
3. Cellular Automaton (Excitable media model) (CA (Excitable media model))
4. Cellular [45]
5. Folding of Texton Placement (Folding_Texton) [36]
6. Folding of Cellular (Folding_Cellular)
7. Folding of Fractal (Folding_Fractal)
8. Folding of Perlin Noise (Folding_Perlin)
9. Fractal (one-over-fBeta-noise) [46]
10. Fractal (Fourier spectral synthesis) [47, 48]
11. Fusion of Cellular and Texton Placement models (Fusion Cellular & Texton) [49]
12. Fusion of Perlin Noise and Cellular models (Fusion Perlin & Cellular)
13. Fusion of Perlin Noise and Texton Placement (Fusion Perlin & Texton)
14. Islamic Patterns [50, 51]
15. Matrix Transformation [52]
16. Perlin Noise [34, 35]
17. Reaction Diffusion [53, 54]
18. Texton Combination with Addition Rules (Texton Addition) [21, 55–57]
19. Texton Placement with Probability Map Rules (Texton probability map)
20. Texton Placement with Random Grid Rules (Texton random grid)
21. Texton Placement with Random Walk Rules (Texton random walk)
22. Texton Placement with Regular Grid Rules (Texton regular)
23. Wavelet Noise [58, 59]

First, for each model, we generated a large number of textures by linearly increasing the value of model parameters so as to sufficiently cover the range of appearances. We treated these textures as surface height maps which were not rendered under any lighting. Their resolution was set at 512*512. Then, we selected samples with obviously different appearances from height maps produced by each model. It should be noted that we simply performed uniform sampling of the whole parameter space. The number of textures chosen from each model varied. It depended on the range of texture surfaces they could generate. Overall we had 450 texture samples that included as much variety of texture types as possible.

**Fig 1:**
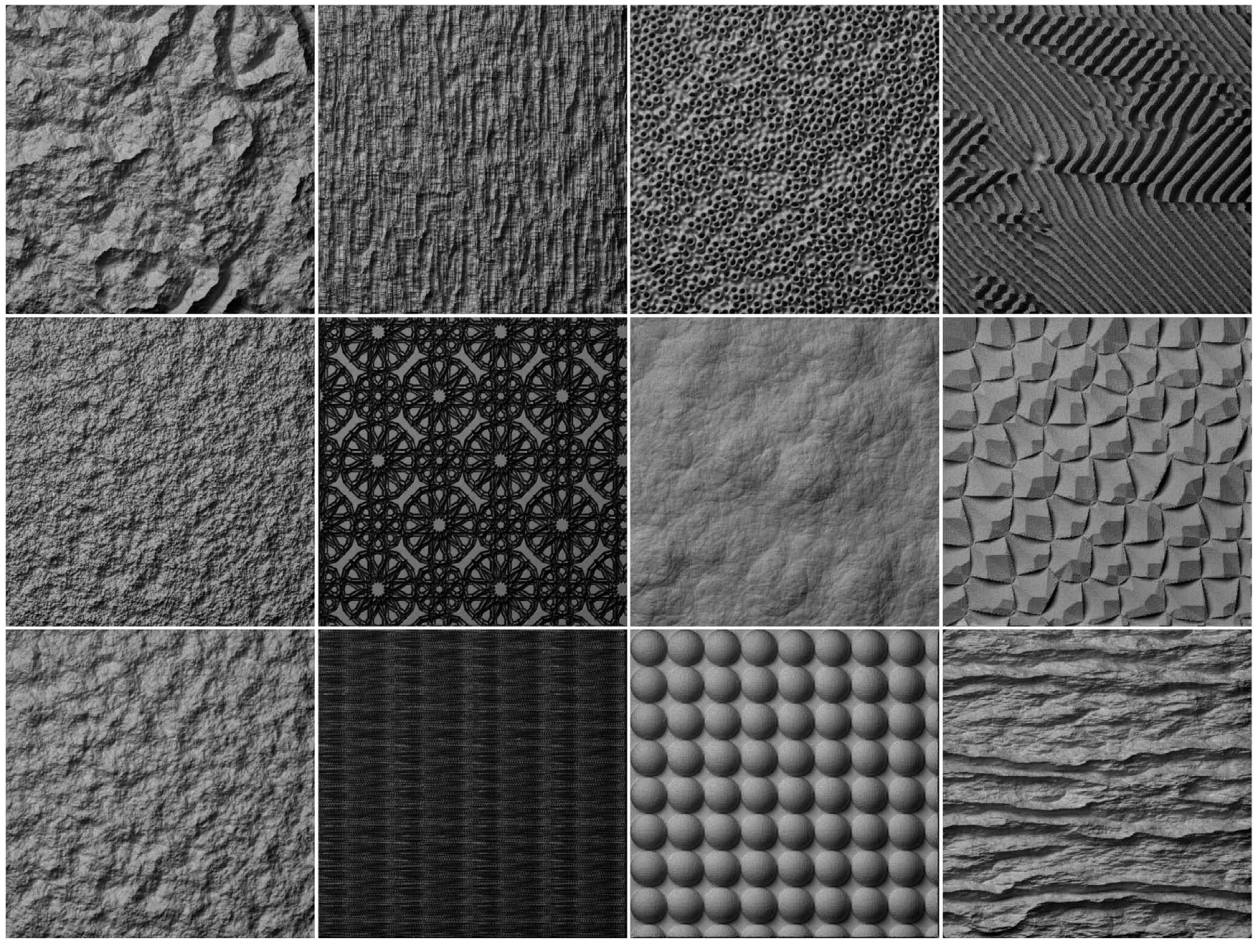
Example samples in the dataset.

In order to understand how humans naturally categorize texture surfaces, it is important to present them in imageries so that they can be envisaged as being of real surfaces [60]. Natural-like textures can be produced by using a ray tracing algorithm, which is able to simulate realistic lighting and a wide range of optical effects, the most important of these being inter-reflections. The resulted effects such as reflections and shadows will produce high degree of visual realism. We employed a physics-based rendering engine—LuxRender. Each height map of textured surface was rendered under Lambertian conditions and constant albedo; and all were rendered at the slant angle of 45° and tilt angle 135°. Each rendered texture was printed on a 4 * 4 inch photographic paper with the resolution of 128 pixels per inch. The advantage of using photos in the experiments was that subjects were able to look through the whole texture dataset, and it was more favorable for subjects to make judgments according to experimental requirements. Fig 1 shows some example textures from our dataset. The full list of samples was included in the supporting information(S1 Fig, S2 Fig, S3 Fig, S4 Fig, S5 Fig, S6 Fig).

It should be noted that all the samples were grey scale textures. Our samples were limited by available procedural models and they did not sufficiently cover all types of natural textures. However, they can still represent a variety of natural appearances.

### Experiments

Two psychophysical experiments were conducted on 450 samples, including free grouping and rating on 9 Likert scales. Because of the large number of texture samples, it was difficult for the subjects to browse all the textures on a table in the grouping and rating experiments. Thus, we divided the samples into sub-groups for progressive free grouping and rating, which also avoided fatigue in the experiments.

#### Experiment 1: Free grouping

In the grouping experiment, subjects were asked to sort textures into groups according to visual similarity. First, the samples were divided into nine sets randomly; each set contained fifty textures, and each time we presented one set of fifty textures to subjects for grouping. The samples were randomly placed on a flat table so that the subject was able to examine all the stimuli. Then the subject was asked to make groups of textures according to their perceived similarity. When one set was finished, another set of fifty textures would be inserted into groups that already existed. The subject may create new groups, split, merge groups, or move textures between groups. The procedure was repeated until all the textures were sorted into groups. Singleton was not allowed to be a group and grouping based on context of the image was discouraged.

When the initial groups were formed, subjects were asked to merge the groups into clusters in which groups shared visual similarity in one or more aspects. The process was repeated until all initial groups were merged into one cluster. Each merging stage was recorded. It should be noted that the free grouping does not necessarily provide metric estimates of similarity.

Based on the groups sorted by each subject, a symmetric 450 * 450 similarity matrix *S*_*tex*_ was constructed. We defined the similarity between two objects *i* and *j* as the numbers of distinct groups in which *i* and *j* were grouped. Then similarity matrices of 20 subjects were averaged to form a similarity matrix *S* with entries ranging from 0 to 1. The entries *s*_*ij*_ close to 1 meant that more subjects sorted the object *i* and *j* in the same group and vice versa.

Similarity of texture generation models *S*_*mod*_ was constructed based on the texture pairs’ similarities. If texture pairs belonging to two different texture generation models had high similarity scores, we believed that these two models were highly similar. The similarity matrix of procedural models was obtained by averaging the texture similarity scores in individual models.

#### Experiment 2: Rating

The main objective of the rating experiment was to determine perceptual scales for each sample. Salient perceptual features for texture generation models were also highlighted by analyzing rating data. The samples were randomly divided into nine sets as in the grouping experiment. Each subject was assigned with two or three sets of samples and asked to rate the samples on 9-point Likert scales. We used a set of 12 perceptual features as defined in [18]. The 12 perceptual features were: in order, contrast, repetition, granularity, randomness, roughness, feature density, directionality, structural complexity, coarseness, regularity, local orientation, and uniformity. Likert scaling was essentially a bipolar scaling method, measuring either positive or negative response to a property. Thus, high values and low values of 9-point Likert scales represented the opposite property of one perceptual feature. The polarity of the 9-point Likert scales was explained to subjects, for example, what the lowest and highest values stand for. Scale 1 for each feature represented low contrast, non-repetitive, non-granularity, non-random, rough, low feature density, non-directional, low structural complexity, coarse, irregular, non-oriented and non-uniform in order. In contrast, scale 9 represented high contrast, repetitive, granularity, random, smooth, high feature density, directional, high structural complexity, fine, regular, locally oriented and uniform. Thus, 24 adjectives that describe textures were given to assess to what extent the features were perceived by subjects for given textures.

A sample-feature matrix *F*_*tex*_ was constructed by averaging the Likert scales of each sample, of which *r*_*ij*_ represents the *j*_*th*_ perceptual features for sample *i*.

For each texture generation model, the model-feature matrix *F*_*mod*_ was obtained by averaging Likert scales of samples produced by the model. It should be noted that this can only be seen as a rough guide for general use because perceptual features of different textures generated by the same model may be slightly different.

## Results

### Identifying features of procedural texture generation models

**Fig 2:**
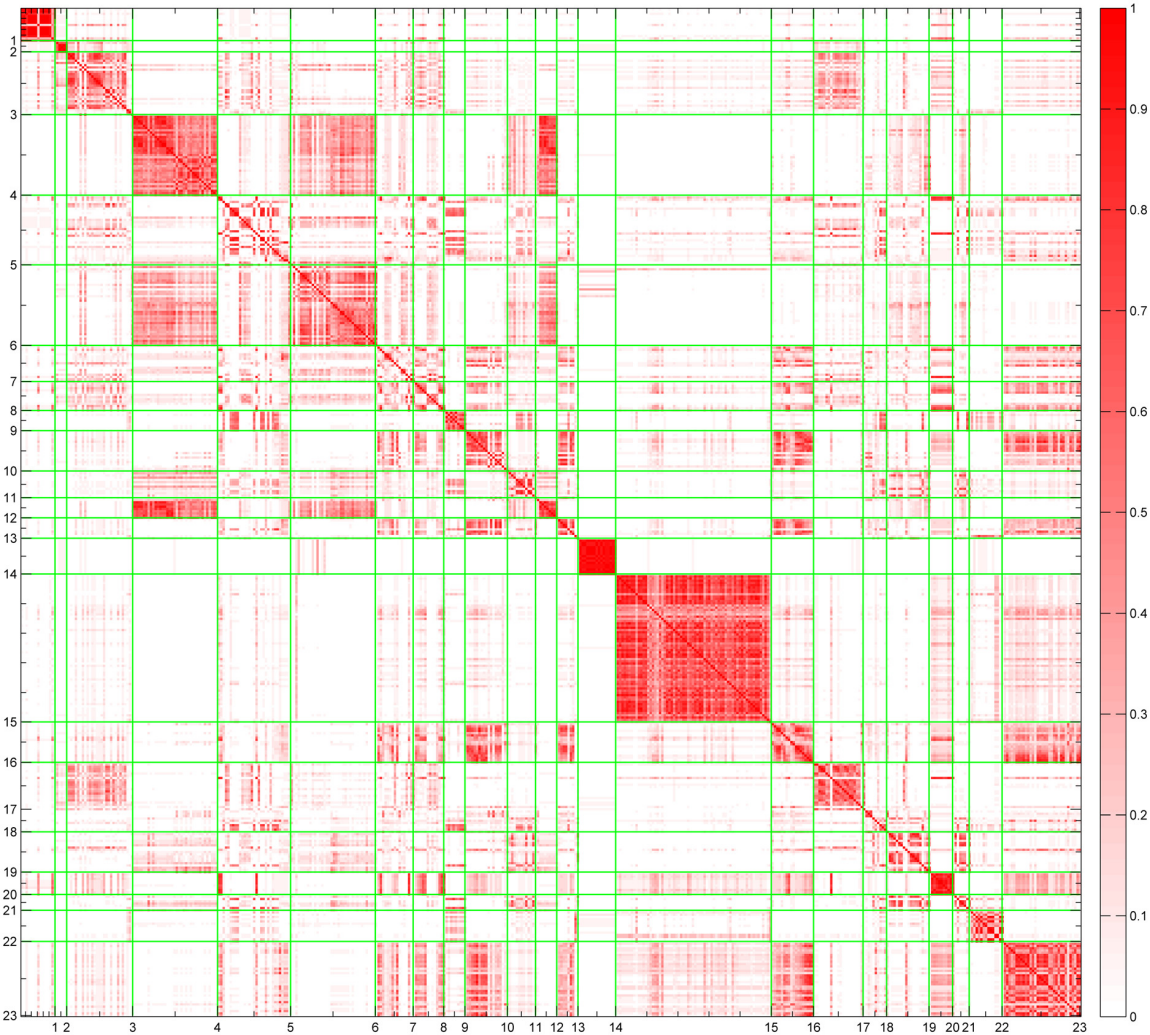
The similarity matrix constructed from grouping experiments. The colors indicate the similarity between pairs of samples as specified by the color bar. The labels on the axes represent the 23 procedural models. The distances between the labels represent the number of samples. The green lines separate samples generated by different procedural models. Point colors in block represent the similarity between samples generated by one certain model to another.

In the free grouping experiment, subjects were asked to group the samples into clusters according to the visual similarity. It was also of our interest to ask subjects the reason that they grouped the samples. Fig 2 shows the averaged similarity matrix obtained from subjective grouping. Each sample pair was color-coded. First, lighter colors in most blocks suggest that textures generated by corresponding models are less similar. For example, textures generated by models of Matrix Transformation (Label 15 in Fig 2) and Cellular (Label 4 in Fig 2) are dissimilar, and the color in the corresponding block is close to white. However, there were still samples created by different models which overlapped to some extent.

Secondly, along the diagonal of the matrix, colors in block represented similarity between samples produced by the same model. Colors in these blocks close to red suggest that most subjects grouped samples generated by the same model into one cluster. Thus, visualization of the similarity matrix revealed that subjects did cluster different samples according to their underlying procedural models, although a few different models are also similar to a certain extent. The findings also suggest that there was a close relationship between perceptual scales of samples and the procedural models which generated the samples. The underlying ways subjects grouped the samples reflect that samples generated by the same model shared some perceptual characteristics. In order to discover what features subjects used for classification, we attempted to identify perceptual features of different models in the next stage.

Hierarchical cluster analysis (HCA) was a widely used data analysis tool which sought to build a tree diagram called dendrogram that successively merges similar groups of points. In our experiment, the input for HCA was the similarity matrix *S*_*mod*_ obtained from the grouping experiment. We chose the Euclidean distance as distance measure and average linkage clustering as the linkage criteria.

**Fig 3:**
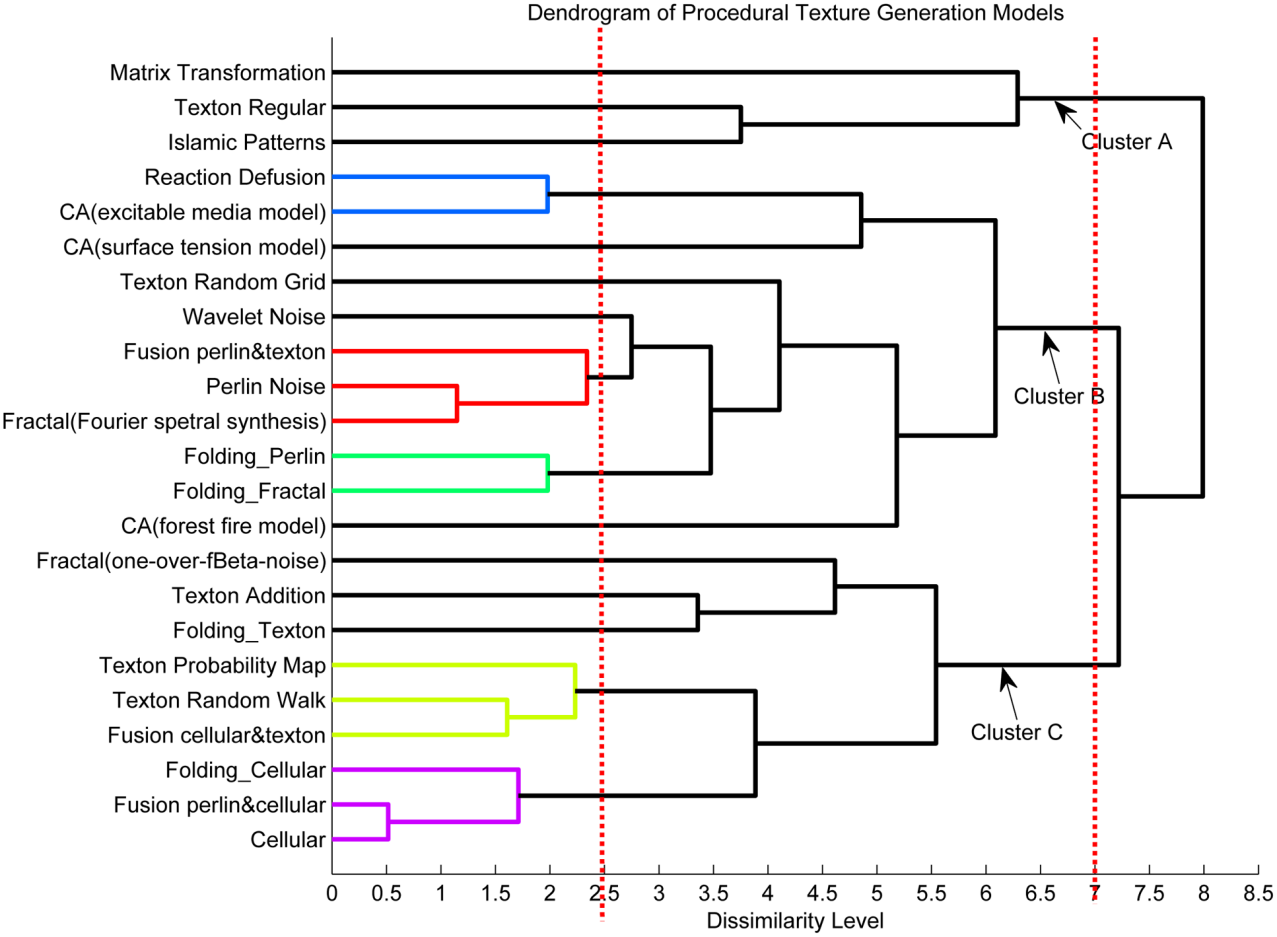
The resulting dendrogram of HCA. Three clusters below the dissimilarity level of 7 were labeled as Cluster A, Cluster B and Cluster C. Models which were classified as groups below the dissimilarity level of 2.5 were represented by different colors.

The resulting dendrogram illustrated in Fig 3 showed how the texture generation models were clustered into different groups. In Fig 3, there were three chunks below a dissimilarity level of 7. Models of “Matrix Transformation”, “Texton Regular” and “Islamic Patterns” were clustered into one group; we named this chunk as Cluster A. However, the “Matrix Transformation” model was separated from the other two models, which meant that textures produced by the “Matrix Transformation” were most dissimilar to others. In fact, the “Matrix Transformation” was a unique method that was capable of generating fabric-like textures which were perceived as uniform, locally oriented and regular. Texture patterns produced by “Islamic Patterns” and “Texton Regular” can be described as regular, directional, and repetitive. In these textures, structural primitives were distributed repetitively and regularly. Cluster B, comprising of 11 models, generated textures with granular textons randomly spread in the images. Tactile roughness appeared in textures generated by models in Cluster B. Cluster C comprised of 9 models, and textures in this category consisted of randomly distributed near-regular shape elements. They shared the features of repetition, near regularity, roughness and coarseness. In this cluster, the “Fractal (one-over-fBeta-noise)” model had large dissimilarity with others, and textures appeared as noticeably vertical stripes. Moreover, models clustered as groups below the dissimilarity level of 2.5 were regarded as being able to generate textures resembling each other. These were marked with different colors in Fig 3.

Although the dendrogram indicated certain similarities between models, characteristics of models can only be inferred from the textures. Thus, we further applied singular value decomposition (SVD) to confirm the perceptual features of these models. SVD was a matrix factorization method closely related to eigenvector decomposition. Recently, SVD had shown extraordinary usefulness in applications of Latent Semantic Analysis (LSA) [61], [62]. Since the feature-by-model matrix obtained from the rating experiment shared certain similarity to the word-by-document matrix in LSA, we also applied SVD to this feature-by-model matrix (*F*_*mod*_) to derive a space representation with reduced dimensions, which were convenient for analyzing any relationships among models and features.

The matrix *F*_*mod*_ was decomposed into the product of three matrices:

$$F_{mod}^{T}=U*S*V^{T}$$ (1)

**Fig 4:**
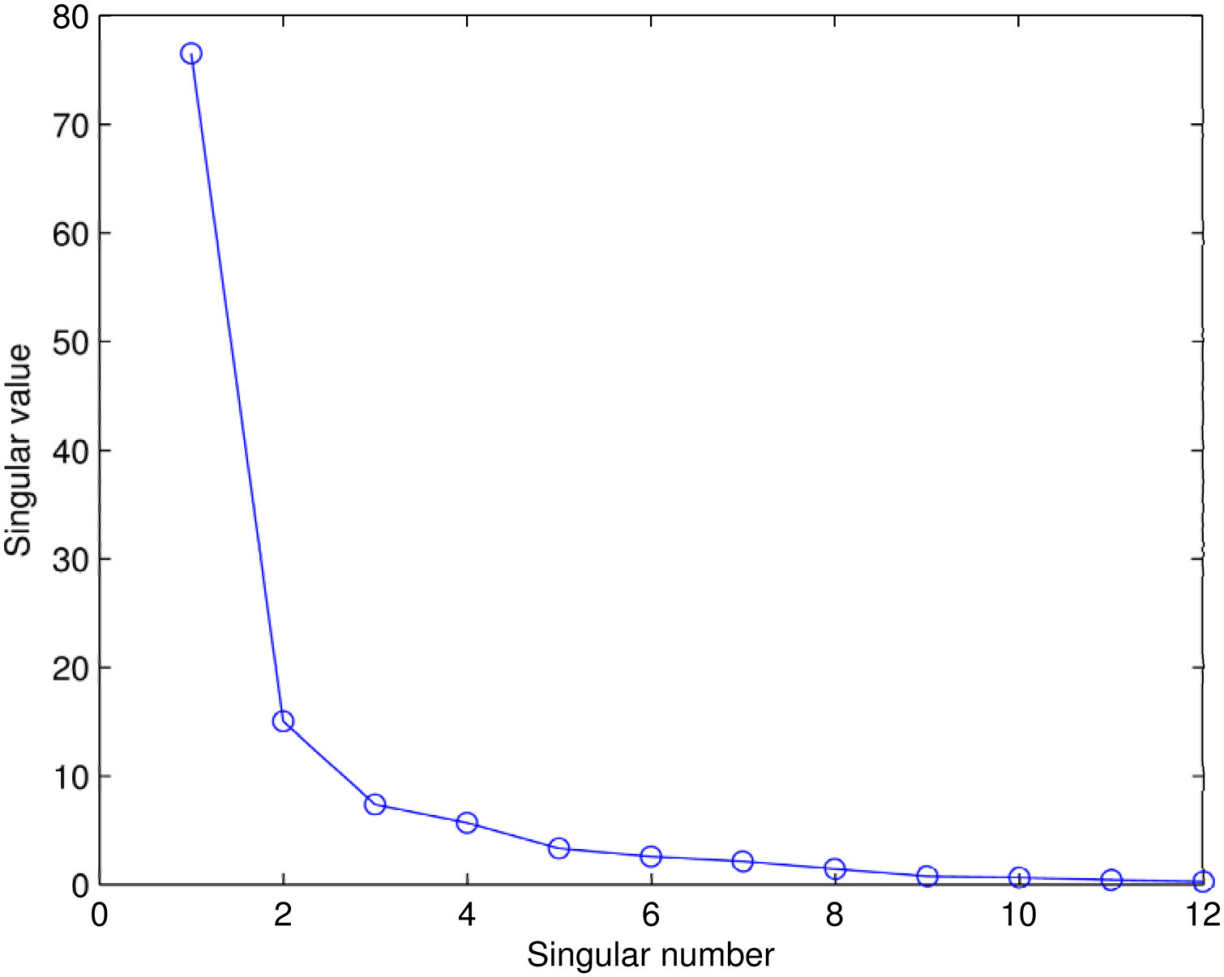
Plot of singular values.

**Fig 5:**
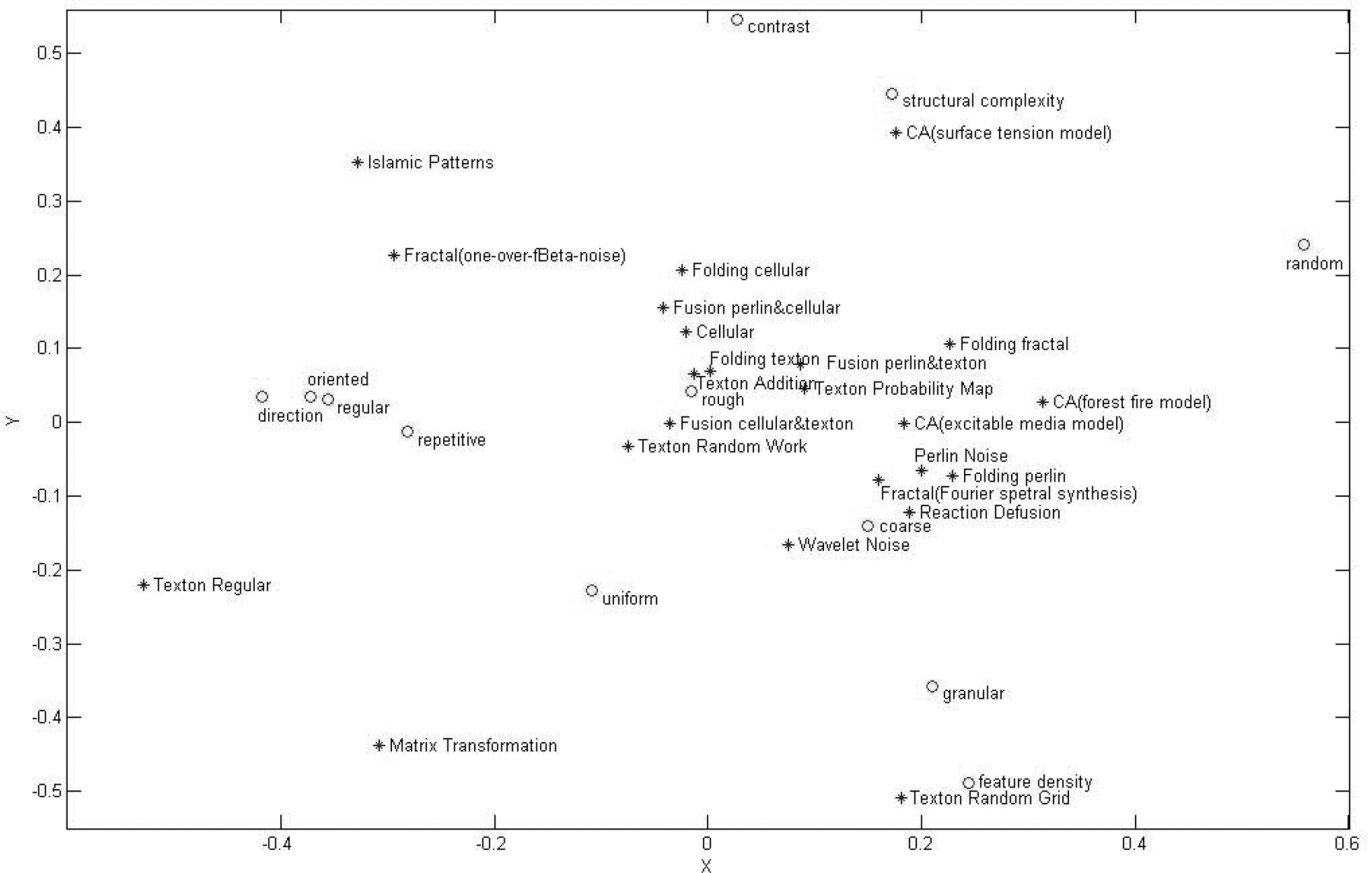
Plot of texture generation models and features. The blue stars represent 23 procedural texture generation models and the red circles represent 12 texture perceptual features.

*U* was the matrix of the eigenvectors of ${\text{F}}_{mod}*{\text{F}}_{mod}^{T}$ which represented the characteristics of models; *V* was the matrix of the eigenvectors of ${\text{F}}_{mod}^{T}*{\text{F}}_{mod}$ which represented the characteristics of features; and *S* was a diagonal matrix of the singular values. We reduced the dimensionality of the solution simply by choosing coefficients in the diagonal matrix, ordinarily starting with the largest. Fig 4 shows the importance that each singular value contributed to the information contained in the original matrix. We kept three singular values and ignored smaller ones, i.e., we reduced *U* and *V* ^*T*^ to *U*_3_ and ${\text{V}}_{3}^{T}$, having 3 columns and rows respectively. Leaving out the first column of *U*_3_ and ${\text{V}}_{3}^{T}$, we plotted the second and third columns on the same graph. The reason we abandoned the first column was that, for models, it corresponded to the numbers of features for each model; for features, it correlated with number of times that features had been used in all models. Thus, it was not informative for our purposes. Fig 5 shows the space representing both features and models. The advantage of this technique was that it was able to not only identify the clusters of models, but also link the features to models by measuring distances between points.

Distances between points correspond to the correlation of features, models or both. Each coordinate represents several features that were responsible for the similarity between models and characteristics of each model. For abscissa, the terms “oriented”, “direction”, “regular”, and “repetitive” were located at the negative side of the axis and were close to each other spatially; it meant that these four features had high positive correlations. Conversely, the term “random” was located on the positive side. The distribution indicates that the four features were negatively correlated with the feature of “random”. Models in Cluster A crowded around these words implied that such characteristics were easily perceived by humans for textures created by these models. Models in Cluster B scattered in the middle of the abscissa. These models produced surfaces with near-regular textons distributed in a random and repetitive way. Points representing these models were close to the point “rough”; thus the apparent perceptual feature was roughness for corresponding textures. Models in Cluster C scattered on the positive side. Textures generated by these models are generally perceived as natural surfaces, e.g., sand and cement. For ordinate, models on the positive side could produce complex structural patterns with high contrast. The typical models with these two features included “Islamic Patterns” and “CA(surface tension model)”. Features including “density”, “granular” and “uniform” appeared at the negative side. Meanwhile, the “Texton Random Grid” and “Matrix Transformation” models at the furthest end of the negative axis implied that textures generated by these two models possessed those features. In general, the results based on SVD confirmed the inference on the features associated with different models.

### Perceptual Texture Space

**Fig 6:**
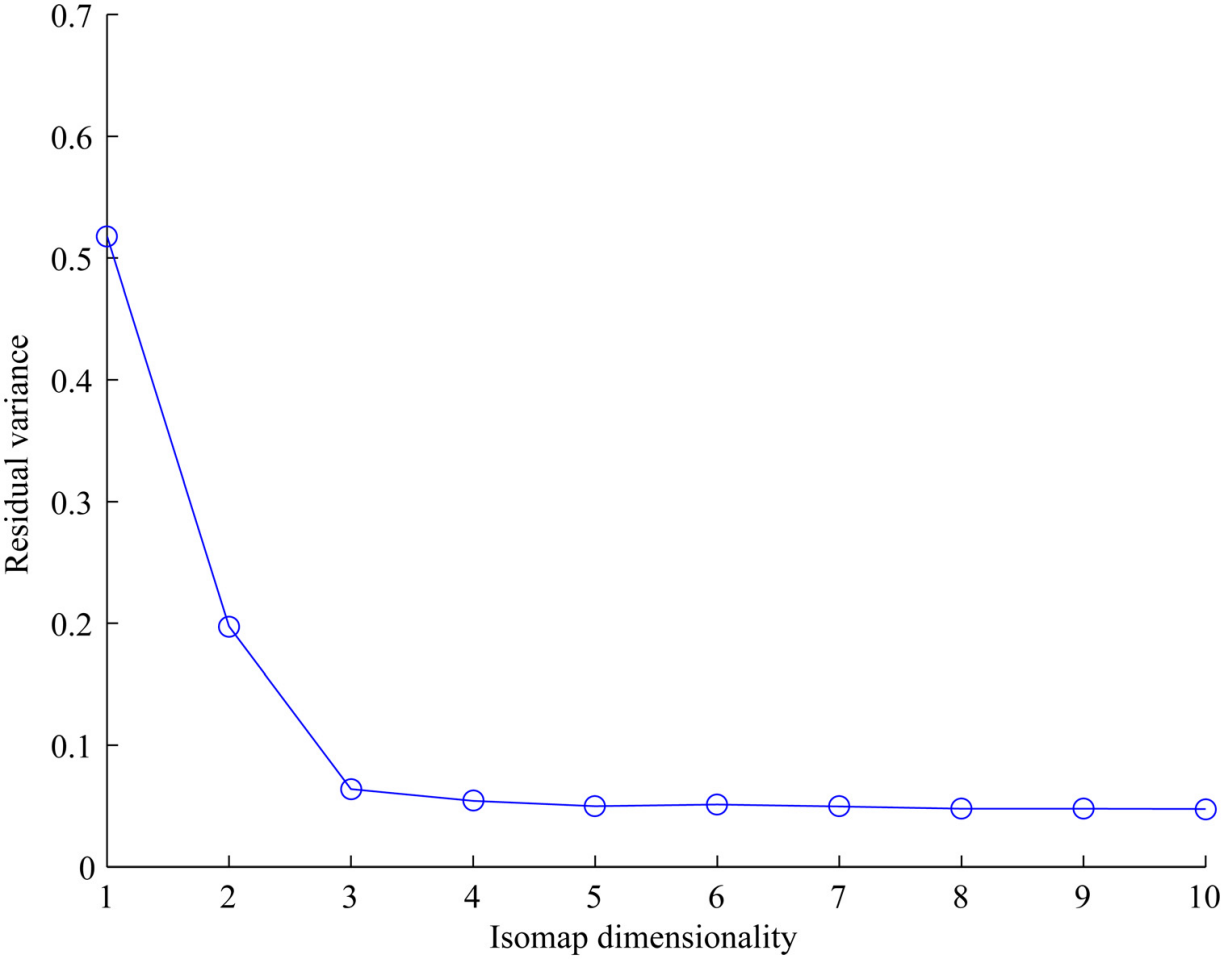
Plot of residual variance and Isomap dimensions.

We further applied the isometric feature mapping algorithm (Isomap) [63] to the texture dissimilarity matrix obtained from subjects’ grouping to derive a perceptual texture space (PTS). Isomap helped to identify the perceptual dimensions in the PTS that subjects used to cluster the textures. The variation of visual texture appearances could be estimated along one axis representing the degree of perceived features. The intrinsic dimensionality was evaluated by computing the residual variance from dimension one to ten in our case. The relationship between residual variance and reduced dimensions is shown in Fig 6. According to the plot in Fig 6, dimensionality can be estimated by looking for the elbow at which this curve ceased to decrease significantly with added dimensions. In our case, three dimensions seemed to be a reasonable choice.

To identify the underlying dimensions of this space, for each dimension, we investigated the relevant features by computing correlations between each coordinate and average scales of samples. We also attempted to verify whether the perceptual dimensions were modulated by stimuli sets. To achieve this goal, eight data sets were tested, of which seven subsets were selected from the original dataset and the remaining one was the full dataset. The corresponding similarity matrices were extracted from the original similarity matrix from the grouping experiment.

For comparison, three dimensions were chosen as the embedded dimensions. Table 1 displays the correlation coefficients between 3 axes (X, Y and Z) of perceptual space and average scales of 12 perceptual features for eight subsets. Axes in the perceptual space for each subset were significantly related to certain perceptual features. As for the axes of perceptual space constructed from all the samples (i.e. subset 8), the X axis was positively correlated with the features of density and coarseness; the Y axis was positively correlated with those of repetitive, rough, direction, regular, oriented and uniform, while negatively correlated with random; and the Z axis was positively correlated with the features of contrast, granular and structural complexity.

**Table 1:** Correlation coefficients between the 3 axes (X, Y and Z) of the perceptual space and the average scales of 12 perceptual features for eight subsets of the original data. Each subset included samples produced by a number of models. Subset 1 to 7 included samples generated by all models except for CA(Forest fire model), Matrix Transformation, texton models, CA models, Cellular, Folding models, folding and fusion models, respectively. Subset 8 contained all samples, i.e. the original data set.

Subset	Axe	contrast	repetitive	granular	random	rough	density	directional	complexity	coarse	regular	oriented	uniform
1	X	-0.27	0.22	0.27	-0.23	-0.01	**0.75**	0.23	-0.24	0.63	0.20	0.22	0.51
	Y	0.12	**0.67**	-0.32	**-0.77**	**0.47**	-0.07	**0.67**	0.01	0.05	**0.76**	**0.70**	**0.52**
	Z	**0.32**	0.29	**0.46**	-0.08	-0.09	0.12	-0.22	**0.38**	0.09	0.15	-0.15	0.14
2	X	-0.13	-0.25	**0.40**	0.33	-0.30	**0.67**	-0.31	-0.03	**0.55**	-0.33	-0.33	0.05
	Y	**0.53**	0.38	0.35	-0.15	-0.10	-0.08	-0.17	**0.52**	-0.10	0.25	-0.11	0.08
	Z	**-0.48**	**-0.51**	0.09	**0.50**	-0.02	-0.02	**-0.67**	0.07	-0.12	**-0.56**	**-0.65**	**-0.41**
3	X	**-0.44**	-0.01	0.34	0.01	0.04	**0.75**	0.07	-0.29	**0.65**	0.00	0.05	0.29
	Y	-0.35	**-0.70**	-0.22	**0.56**	-0.19	-0.15	-0.32	-0.37	-0.20	**-0.63**	-0.38	**-0.48**
	Z	-0.32	**0.43**	**-0.50**	**-0.64**	**0.44**	0.07	**0.70**	-0.24	0.12	**0.58**	**0.69**	**0.47**
4	X	-0.37	0.29	0.15	-0.37	0.19	**0.73**	**0.40**	**-0.41**	**0.67**	0.32	0.40	**0.59**
	Y	-0.30	**-0.43**	**-0.42**	0.27	0.03	-0.24	0.08	**-0.44**	-0.20	-0.31	0.00	-0.32
	Z	0.28	**0.62**	-0.35	**-0.71**	0.35	-0.23	**0.71**	0.10	-0.10	**0.72**	**0.71**	0.38
5	X	**0.48**	-0.10	-0.16	0.18	-0.17	**-0.70**	-0.19	**0.40**	**-0.61**	-0.13	-0.18	-0.39
	Y	-0.14	**-0.72**	0.36	**0.82**	**-0.43**	0.04	**-0.78**	-0.01	-0.09	**-0.81**	**-0.80**	**-0.59**
	Z	0.35	0.32	**0.42**	-0.13	-0.06	0.15	-0.14	**0.40**	0.13	0.20	-0.08	0.17
6	X	0.39	-0.20	-0.25	0.23	-0.09	**-0.78**	-0.22	0.26	**-0.70**	-0.22	-0.24	**-0.46**
	Y	**-0.53**	**-0.56**	-0.21	**0.43**	-0.11	-0.07	-0.29	-0.37	-0.12	**-0.52**	-0.31	-0.33
	Z	0.08	**-0.58**	**0.41**	**0.72**	**-0.55**	0.12	**-0.76**	0.22	-0.02	**-0.70**	**-0.76**	**-0.50**
7	X	**-0.55**	0.10	0.11	-0.16	0.13	**0.66**	0.32	**-0.50**	**0.62**	0.15	0.32	0.35
	Y	-0.22	0.12	**-0.52**	-0.31	**0.43**	**-0.41**	0.40	**-0.45**	-0.27	0.23	0.38	0.11
	Z	0.26	**0.79**	0.26	**-0.69**	0.34	0.18	**0.67**	-0.07	0.22	**0.77**	**0.67**	**0.60**
8	X	0.35	-0.12	-0.28	0.15	-0.08	**-0.74**	-0.17	0.30	**-0.66**	-0.13	-0.17	-0.40
	Y	-0.13	**-0.70**	0.36	**0.80**	-0.40	0.03	**-0.77**	0.02	-0.10	**-0.79**	**-0.79**	**-0.58**
	Z	**0.48**	0.32	**0.41**	-0.11	-0.14	0.09	-0.12	**0.48**	0.07	0.19	-0.06	0.13

Correlation coefficients varied with axes and subsets relating to different perceptual features. For subset 1, the correlation between the feature “random” and the Y axis of the perceptual space was negative (-0.77). By contrast, “random” was significantly positively correlated with the Z axis and the correlation coefficient decreased to 0.50 for subset 2. Surprisingly, although the correlations between axes and features varied among the subsets, the combination of features related to different axes seemed unchanged.

**Fig 7:**
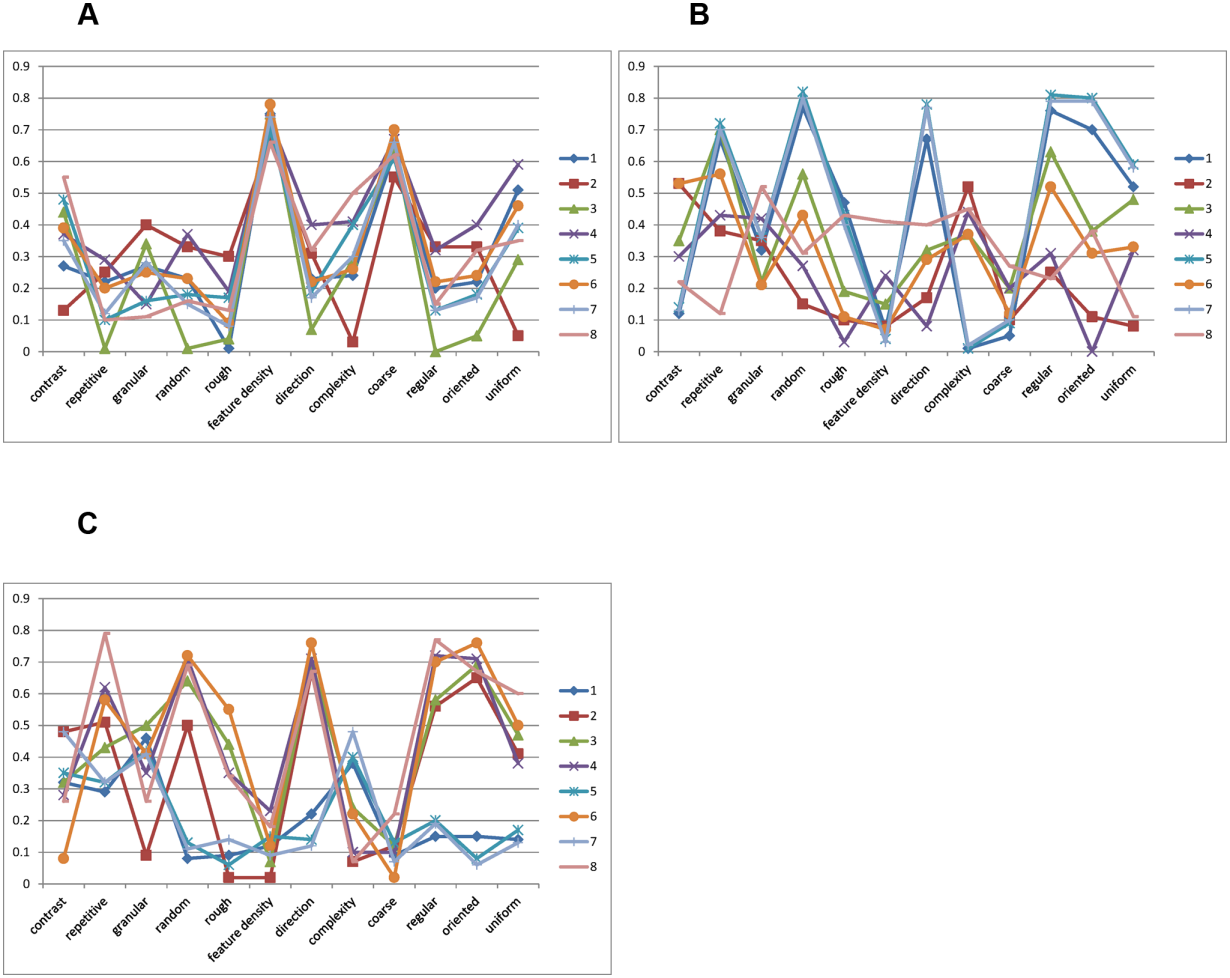
Magnitude of correlation coefficients between the 3 axes ((A) X, (B) Y and (C) Z) in the perceptual texture space and the average scales of 12 perceptual features for eight subsets.

To gain insight into the perceptual dimensions for different subsets, we plotted the correlation of all 8 subsets for each axe without considering the polarity of correlation. As can be seen in Fig 7a, for axis X, the significant correlation occurred at the features “feature density” and “coarse” for all the subsets. In Fig 7b, for subsets 1, 5 and 7, axis Y was significantly correlated with the features “repetitive”, “random”, “direction”, “regular”, “oriented” and “uniform”. For others, the correlations were not obvious, but it seemed that “contrast” and “structural complexity” were the common features related to axis Y. In Fig 7c, for subsets 1, 5 and 7, axis Z correlated with the features “contrast”, “granular” and “structural complexity”, while the others significantly correlated with the features “repetitive”, “random”, “direction”, “regular”, “oriented” and “uniform”.

The results suggest that axes correlated with combinations of features, although the weights of combination for different dimensions varied with stimuli. The three combinations were “feature density” and “coarse”; “repetitive”, “random”, “direction”, “regular”, “oriented” and “uniform”; “granular” and “structural complexity”. It was interesting to find that the dimensions we identified were similar to Rao’s [18], in spite of the fact that the stimuli and subjects in the two studies were different. Overall, the feature combinations corresponding to individual dimensions remained unchanged; however, the significance of the three dimensions in the perceptual texture spaces varied with different sets of stimuli.

**Fig 8:**
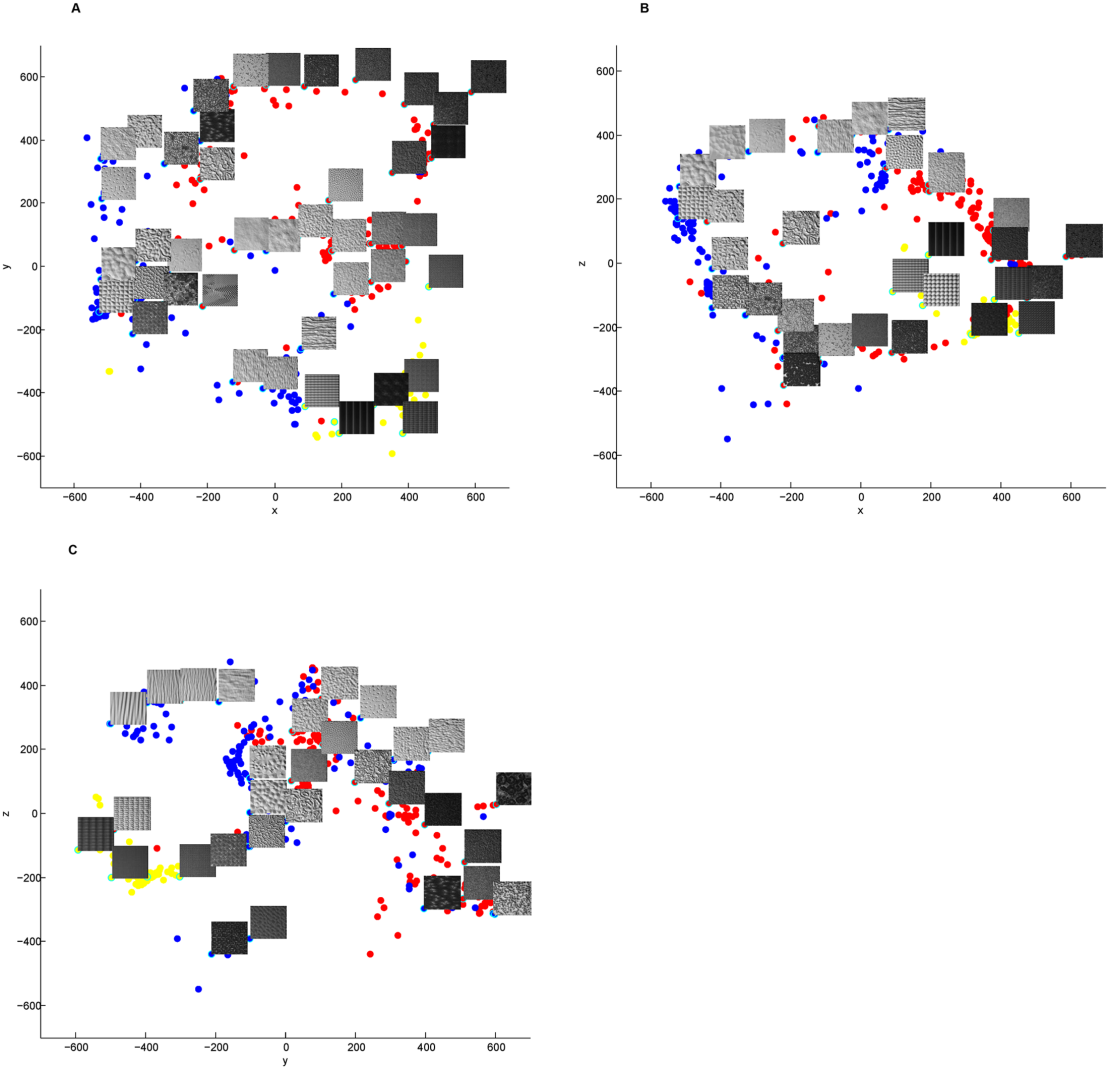
Three dimensional representation of the Perceptual Texture Space based on Isomap. The projection into the (A) x-y plane, (B) x-z plane, (C) y-z plane were shown. Points labeled with yellow, red and blue corresponded to Cluster A, Cluster B and Cluster C resulted by HCA respectively.

Fig 8 exhibited two-dimensional projections produced by Isomap. Selected samples were superimposed on the data points. Along certain axis, textures can be perceived similar or different according to visual perceptual features. We did not interpret the dimensions as Rao [18] did, for we believed that the dimensions were difficult to describe and further psychophysical experiments were needed to assess the underlying dimensions. As shown in Fig 7, correlation analysis suggests that subjects used a combination of perceptual features as criteria while grouping textures.

The perceptual texture space was constructed based on the dissimilarity matrix by using the Isomap algorithm derived from eigen decomposition. However, this texture space could only provide an embedding for given training points. There was no straightforward extension to out-of-sample examples because it was impractical to re-compute the eigenvectors. In terms of our method, it was impossible to conduct a grouping experiment again when given out-of-sample examples. In order to simulate the mechanism that subjects used to judge the similarity of textures pairs and extend the method to out-of-sample examples, appropriate computational models would be necessary for integrating perceptual features.

Regression analysis was used to find a set of functions *f*_*k*_(*r*)(*k* = 1, 2, 3) that mapped the perceptual feature vectors *r* of each texture to the coordinates *p*_*k*_ of PTS, i.e. *p*_*k*_ = *f*_*k*_(*r*)(*k* = 1, 2, 3). The regression was typically nonlinear and three SVM regressions were used to determine the nonlinear mapping models. The three dimensions in the PTS were highly correlated with a certain number of perceptual texture features, instead of all 12 features. Accordingly, a subset of feature vectors was selected based on the correlation results for each dimension. Altogether, three subset feature vectors *r*_*i*_(*i* = 1, 2, 3) were formed as input training features to SVM regression models and three models were trained. Table 2 shows the performance of the three regression models evaluated by mean squared errors(MSE) and squared correlation coefficients. Thus, when given a set of out-of-sample perceptual scales, we can accurately predict the intrinsic representations in the PTS by using regression models.

**Table 2:** Mean squared errors and squared correlation coefficients produced by regression models.

PTS	Mean Squared Error	Squared Correlation Coefficient
X	0.0047	0.9425
Y	0.0079	0.8919
Z	0.0057	0.8972

### Texture generation based on perceptual dimensions

Though texture perception has been studied in image retrieval and classification research in recent years, efforts in texture generation and synthesis based on visual perception are somewhat unnoticed. The purpose of our work was to find a procedural texture generation model which was capable of generating texture with given perceptual features.

First, we had to inspect whether the 12 perceptual features were sufficient to discriminate different categories of textures. The HCA results of our previous analysis suggested that models clustered into groups at the low dissimilarity level always generated similar or even the same textures. Thus, we merged models at the dissimilarity level of 2.5 according to the HCA results.

We used Support Vector Machines (SVM) to predict procedural models that can generate textures with given perceptual scales. We employed the twelve perceptual features and combinational features learned in the PTS respectively, and conducted leave-one-out cross-validation tests for three perceived clusters derived from HCA and the full texture set. Comparison results were provided in Table 3.

**Table 3:** Comparisons of classification accuracy based on using the 12 perceptual features and combinational features learned in the PTS. Numbers in brackets represented corresponding models that were classified as one group.

Features	Cluster A	Cluster B	Cluster C	Full set
	14, 15, 22	1, 2, (3, 17), 20, 23, (7, 8), (10, 13, 16)	9, 18, 5, (19, 21, 11), (6, 12, 4)	
PTS dimensions	98.95%	81.70%	85.12%	82%
12 perceptual scales	98.95%	75.27%	79.76%	76.44%

We noted that the prediction accuracy improved about 6% when using the PTS dimensions (i.e. combinational features) as training features. Furthermore, different accuracies were achieved for three texture categories. Prediction accuracy for Cluster A (regular, periodicity and structured textures) was the highest, followed by Cluster C (random distribution and structured texture) and Cluster B(random textures).

We also calculated the correlation coefficient between the similarity matrix derived from the grouping experiment and other two forms of the feature matrix. The correlation coefficient (r) between the similarity matrix and the Euclidean distance matrix calculated from subjective rating features is 0.3845, while the correlation coefficient between the similarity matrix and the Euclidean distance matrix calculated from PTS dimensions is 0.7306. The results suggested that similarity measure in PTS was more consistent with human perception.

## Discussion

This work studies visual perception of procedural textures and identifies the most important perceptual dimensions. Results of free grouping for 450 procedural textures reveal that subjects do cluster different samples according to the underlying models, although they have no knowledge of the procedural models. In addition, a low dimensional space was constructed; correlation analysis suggests that 12 perceptual features are combined and mapped to three principle dimensions in this space. This indicates that human observers use combinations of perceptual features, instead of individual ones, when judging visual similarity of textures. In addition, the weight of each perceptual feature to form three dimensions in the space varied with stimuli we tested, although the major features corresponding to individual dimensions remained the same.

It is interesting to note that it is trivial for experienced computer graphics users or artists to select a procedural model that can generate a given example image. This is because they appeal to the prior knowledge or experience of texture properties that certain procedural models could produce. In other words, a model may exist in the human mind and it can relate the procedural texture generation methods to perceptual features for the given exemplar. As suggested in earlier work by [12], high-level perceptual features may be related to low-level features, which can be calculated using computational approaches. A statistical model proposed by Simoncelli and Portilla [7] has been successfully used for texture analysis and synthesis; they argue that raw coefficient statistics characterize the perceptual features of regularity, and cross-correlation of scales and orientations play an important role in visual texture patterns. This also implies that the visual system recognizes the perceptual texture characteristics as combinations of lower-level features, such as frequency and scale. In the past, researchers attempted to relate computational representations to several perceptual texture features (e.g. Tumara et al [10, 14–17]), and their results based on certain texture databases did show effectiveness in texture classification and similarity judgment.

Textures generated by the same procedural model have relatively similar appearances, only with slight differences in one or a few perceptual features. Considering the grouping task itself, although there were many different criteria for grouping the textures, subjects tended to classify textures generated by one model as one group. Consequently, perceptually salient characteristics of textures generated by individual models became the main cause for grouping. It is important to note that although twelve perceptual features we tested in the psychophysical experiments were carefully selected, it could not be effectively used to classify textures into certain models as subjects did. The analysis result based on SVD shows that distributions of different models, in terms of textures that they can generate, overlap to certain extents. In other words, samples generated by different models can be similar to greater or lesser extents in the 12-dimensional feature space. Moreover, classification results prove that although the 12 perceptual features are necessary for texture representation and discrimination, they are certainly not sufficient. Specifically, we noticed that they are proved to be useful for regular textures (Cluster A), producing a near 100% accurate classification. In contrast, for the other two of our texture categories (Clusters B and C), it appears that the 12 perceptual features are not sufficient for texture discrimination, especially for random textures. Additional perceptual features should be exploited so that we can reveal the difference between random textures. Altogether, these findings suggest that there exists a relationship between perceptual characteristics and procedural models; however, the relationship is complex and cannot be simply described in the way of a look-up table.

Comparison of classification results as in Table 3 shows that subjects employ perceptual dimensions in the PTS; and these dimensions are related to a combination of perceptual features. Intuitively, discrimination of apparently dissimilar textures is effortless for subjects, because texture representations based on perceptual dimensions are different. However, for textures with visual appearances similar to each other, similarity judgments may be varied with subjects and stimuli. This is because similar textures normally have almost identical scales in predominant perceptual dimensions but different ones in other dimensions, and individual subjects may apply different weights to perceptual dimensions for similarity judgment.

Recently, some works assessed the effect of lighting and environmental conditions on the perception of material perception [64, 65] and interaction of material properties such as gloss and roughness with color appearance [21, 66, 67]. The results suggest that material properties are affected by scene illumination. In our study, we only refer to grey scale textures; whether they can be generalized to the more common definition of visual texture which involves pigmentation variations, is an issue which requires further study.

As a caveat, we must mention that our study is limited to the specific procedural models and perceptual characteristics that we have tested. Our dataset consists of 450 textures generated by 23 procedural models; they did not sufficiently cover the full range of appearances produced by procedural models. Moreover, verbal labels we tested were not sufficient to characterize all possible properties of natural textures. Different perceptual dimensions of texture akin to different color spaces (i.e. RGB and HSV) may exist. Verification of our findings across a large set of procedural textures and perceptual features is preferred.

Perception of procedural textures leads to a better understanding of how procedural models work and how to design them. With the understanding of perceptual properties for different procedural models, users will be able to choose procedural models and produce a desired perceptual appearance. In addition, research into texture perception is useful in understanding the nature of human perception and is important in applications such as estimating material properties, material categorization and evaluation of visual complexity.

## Conclusion

We have used procedural textures as samples for identifying perceptual dimensions that humans use to discriminate textures. Based on extensive psychophysical experiments with a procedural texture dataset, we have established a perceptual texture space in which three dimensions represent combinations of perceptual features that the human visual system uses. Moreover, we found that the weights in the combination of features for different dimensions in the PTS varied with stimuli, while the feature combinations corresponding to individual dimensions remained unchanged. Comparison of classification performances for different texture clusters suggested that 12 perceptual scales were not sufficient for texture discrimination, especially for random textures. Besides, similarity measure in the PTS was more consistent with human perception. In addition, computational models were constructed for mapping perceptual scales to the perceptual space through nonlinear transformation. Finally, we used perceptual dimensions in the PTS as features to predict a texture generation model that can produce texture with user-defined perceptual scales. We hoped to bridge the gap between the communication of visual texture perception, computer graphics and machine vision.

It should be noted that the proposed framework is general and open to all types of procedural models. When more procedural models are added, the training database will be enlarged and the consistency and reliability of the learned PTS will be improved accordingly.

## Supporting Information

S1 Fig The list of samples generated by procedural models used in the psychophysical experiments. (A)Cellular Automaton(Forest fire model)(CA(Forest fire model)) (B) Cellular Automaton(Surface tension model)(CA(Surface tension model)) (C) Cellular Automaton(Excitable media model)(CA(Excitable media model)).
(TIF)

S2 Fig The list of samples generated by procedural models used in the psychophysical experiments. (D) Cellular (E)Folding of Texton Placement(Folding Texton) (F) Folding of Cellular(Folding Cellular).
(TIF)

S3 Fig The list of samples generated by procedural models used in the psychophysical experiments. (G) Folding of Fractal(Folding Fractal) (H) Folding of Perlin Noise(Folding Perlin)(I) Fractal(one-over-fBeta-noise) (J)Fractal(Fourier spectral synthesis) (K) Fusion of Cellular and Texton Placement models(Fusion Cellular&Texton).
(TIF)

S4 Fig The list of samples generated by procedural models used in the psychophysical experiments. (L)Fusion of Perlin Noise and Cellular models(Fusion Perlin&Cellular) (M)Fusion of Perlin Noise and Texton Placement(Fusion Perlin&Texton) (N) Islamic Patterns (O) Matrix Transformation.
(TIF)

S5 Fig The list of samples generated by procedural models used in the psychophysical experiments. (P) Perlin Noise (Q) Reaction Defusion (R)Texton Combination with Addition Rules(Texton Addition) (S) Texton Placement with Probability Map Rules(Texton probability map) (T)Texton Placement with Random Grid Rules(Texton random grid).
(TIF)

S6 Fig The list of samples generated by procedural models used in the psychophysical experiments. (U) Texton Placement with Random Walk Rules(Texton random walk) (V) Texton Placement with Regular Grid Rules(Texton regular) (W) Wavelet Noise
(TIF)
