# Supplementary figures and images for: Visual Perception of Procedural Textures: Identifying Perceptual Dimensions and Predicting Generation Models
Source: PLoS One. 2015 Jun 24;10(6):e0130335. doi: 10.1371/journal.pone.0130335 (PMC4481328; doi:10.1371/journal.pone.0130335)

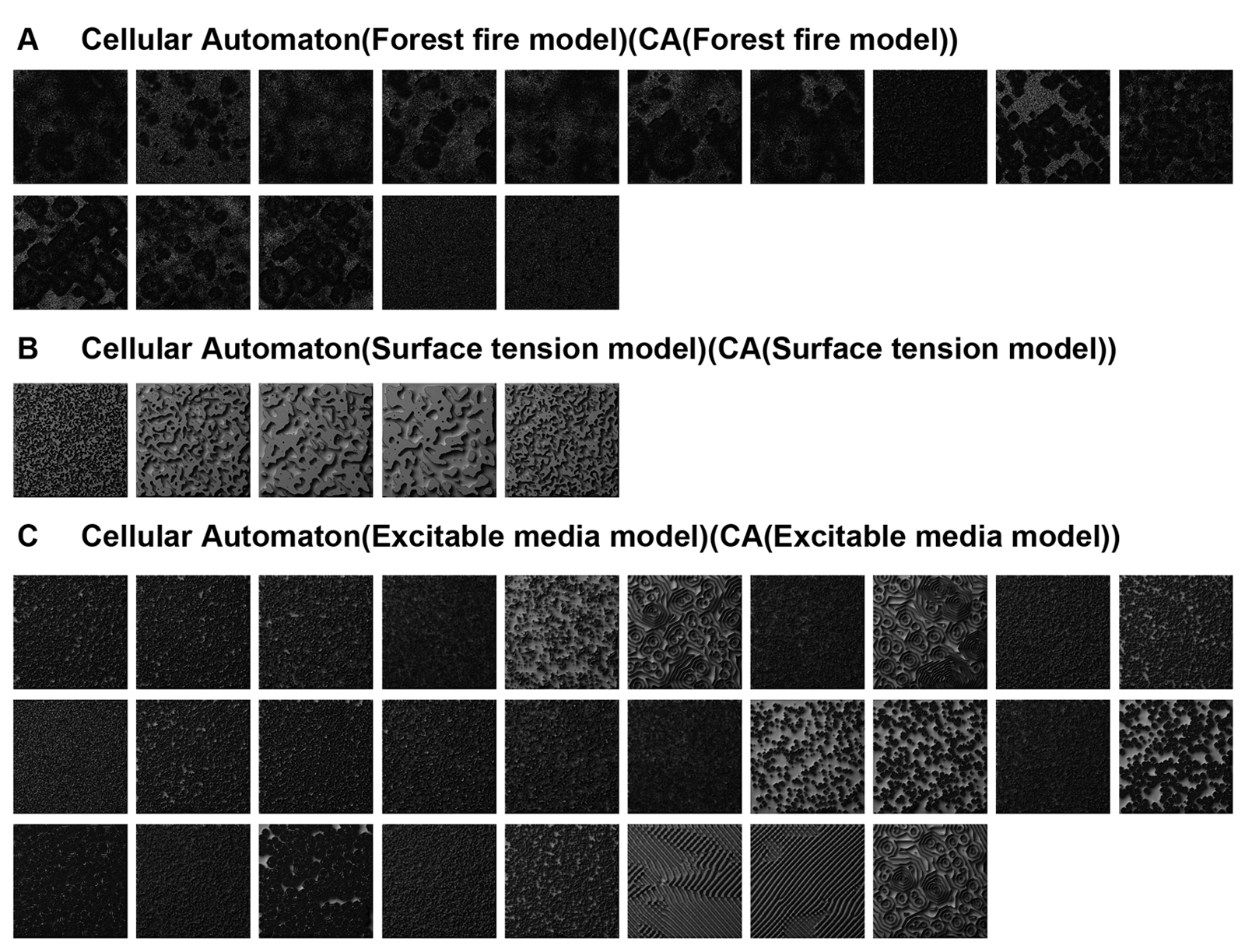

Supplement: S1 Fig — (A)Cellular Automaton(Forest fire model)(CA(Forest fire model)) (B) Cellular Automaton(Surface tension model)(CA(Surface tension model)) (C) Cellular Automaton(Excitable media model)(CA(Excitable media model)). (TIF) [file pone.0130335.s001.tif]

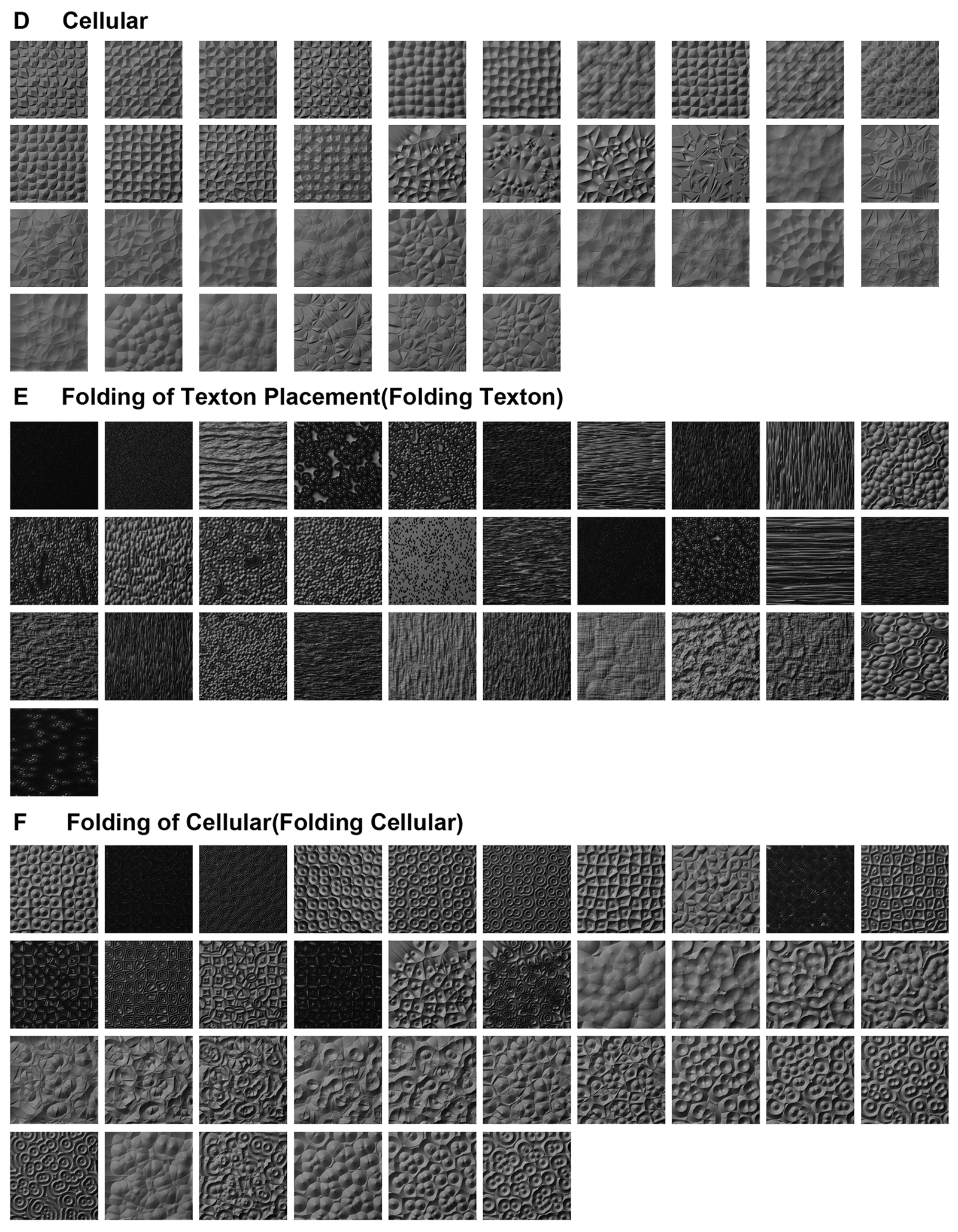

Supplement: S2 Fig — (D) Cellular (E)Folding of Texton Placement(Folding Texton) (F) Folding of Cellular(Folding Cellular). (TIF) [file pone.0130335.s002.tif]

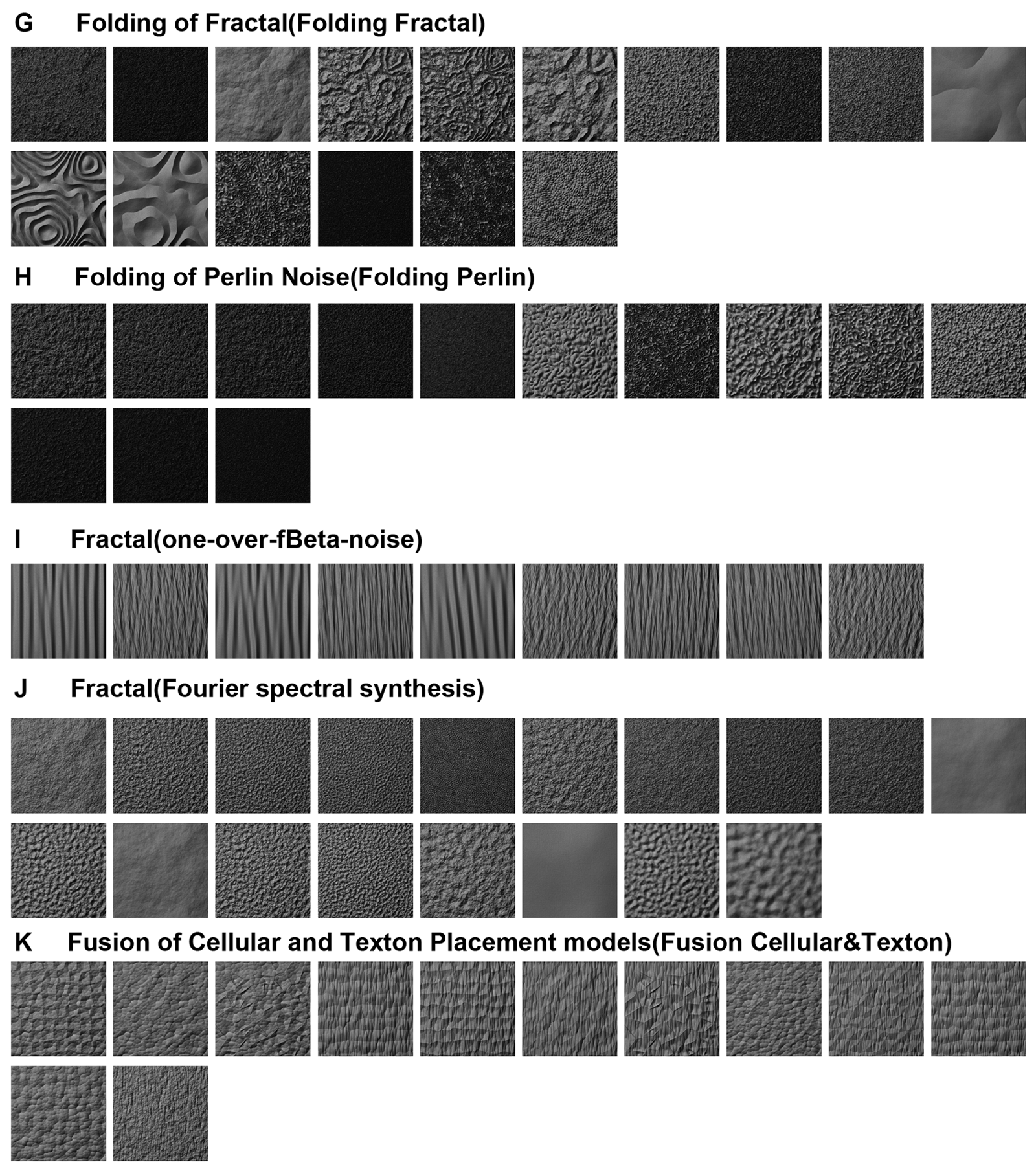

Supplement: S3 Fig — (G) Folding of Fractal(Folding Fractal) (H) Folding of Perlin Noise(Folding Perlin)(I) Fractal(one-over-fBeta-noise) (J)Fractal(Fourier spectral synthesis) (K) Fusion of Cellular and Texton Placement models(Fusion Cellular&Texton). (TIF) [file pone.0130335.s003.tif]

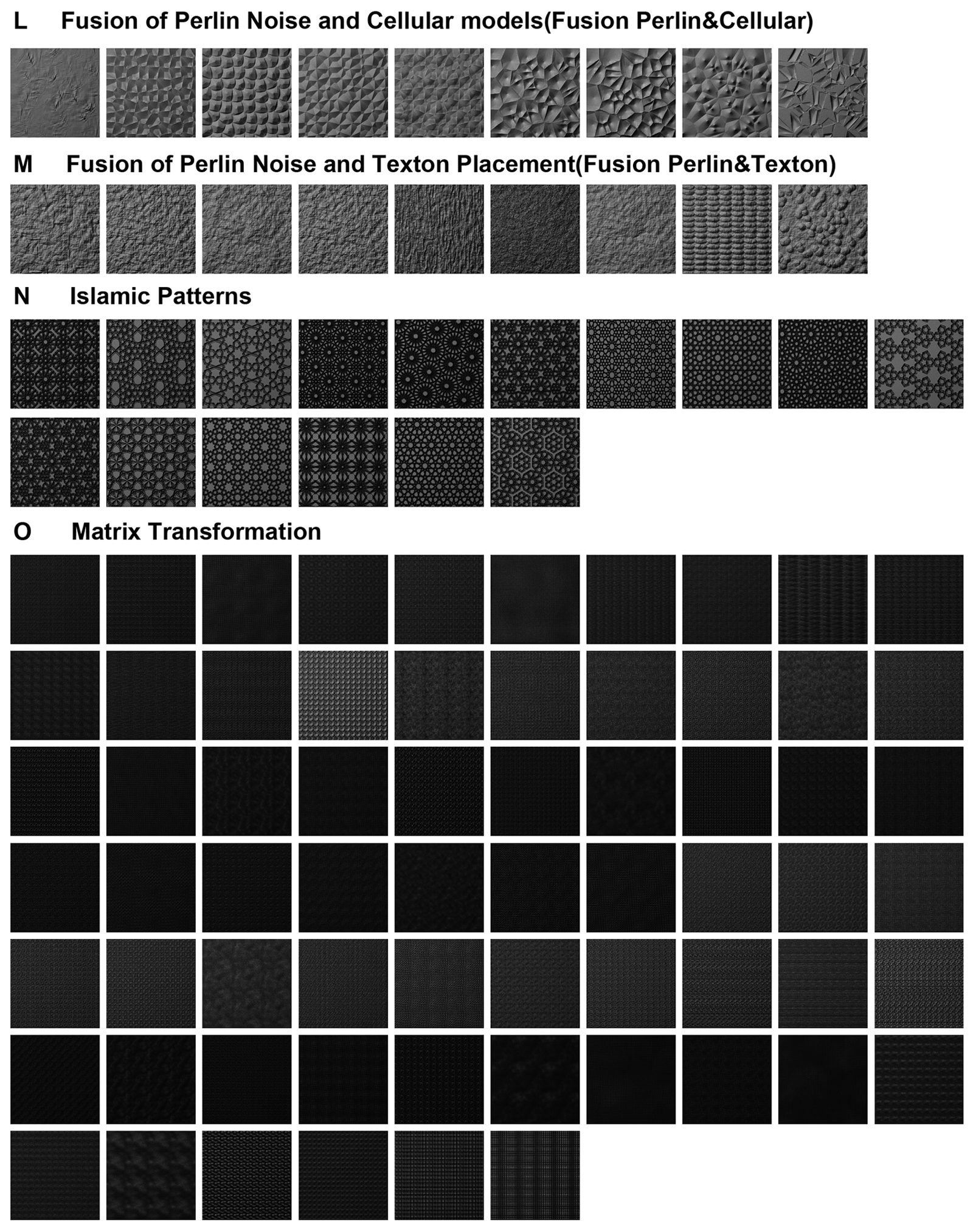

Supplement: S4 Fig — (L)Fusion of Perlin Noise and Cellular models(Fusion Perlin&Cellular) (M)Fusion of Perlin Noise and Texton Placement(Fusion Perlin&Texton) (N) Islamic Patterns (O) Matrix Transformation. (TIF) [file pone.0130335.s004.tif]

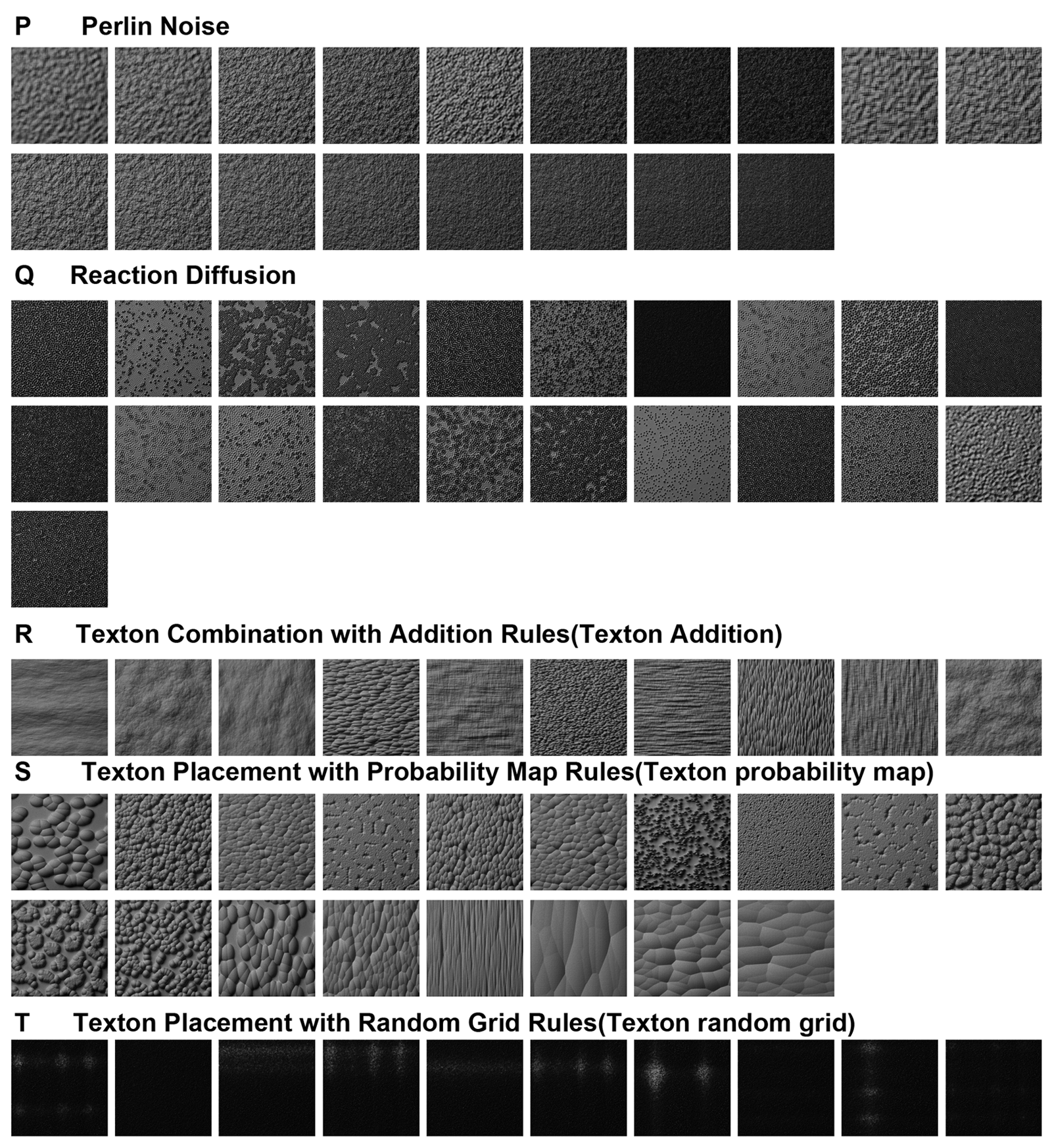

Supplement: S5 Fig — (P) Perlin Noise (Q) Reaction Defusion (R)Texton Combination with Addition Rules(Texton Addition) (S) Texton Placement with Probability Map Rules(Texton probability map) (T)Texton Placement with Random Grid Rules(Texton random grid). (TIF) [file pone.0130335.s005.tif]

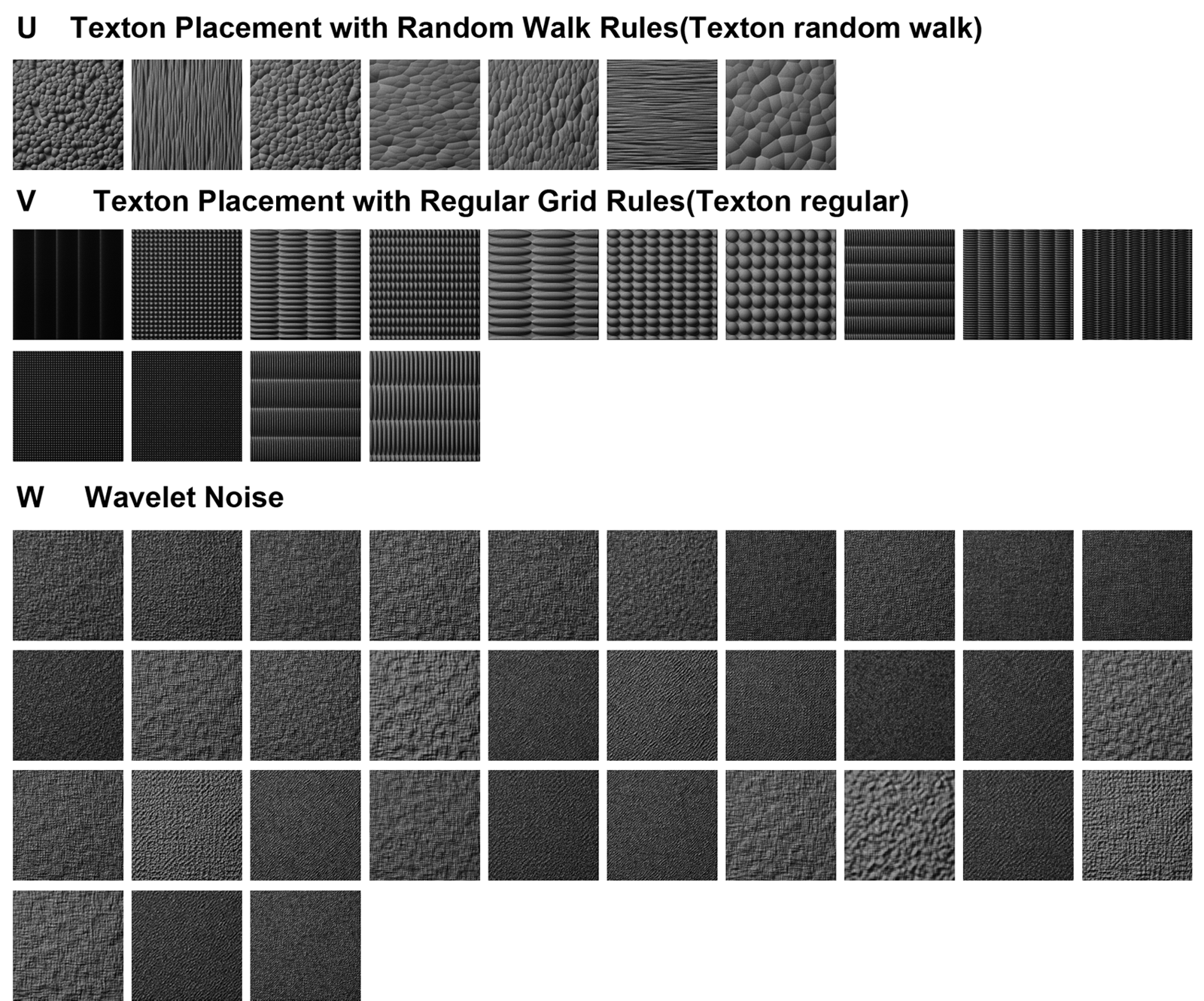

Supplement: S6 Fig — (U) Texton Placement with Random Walk Rules(Texton random walk) (V) Texton Placement with Regular Grid Rules(Texton regular) (W) Wavelet Noise (TIF) [file pone.0130335.s006.tif]
